# That person is now with or without a mask: how encoding context modulates identity recognition

**DOI:** 10.1186/s41235-022-00379-5

**Published:** 2022-04-01

**Authors:** Teresa Garcia-Marques, Manuel Oliveira, Ludmila Nunes

**Affiliations:** 1 ISPA – Instituto Universitário, William James Center for Research, Rua Jardim do Tabaco, 34, 1149-041 Lisbon, Portugal; 2 Association for Pschology Science, Washington, USA

## Abstract

Previous research has mostly approached face recognition and target identification by focusing on face perception mechanisms, but memory mechanisms also appear to play a role. Here, we examined how the presence of a mask interferes with the memory mechanisms involved in face recognition, focusing on the dynamic interplay between encoding and recognition processes. We approach two known memory effects: (a) matching study and test conditions effects (i.e., by presenting masked and/or unmasked faces) and (b) testing expectation effects (i.e., knowing in advance that a mask could be put on or taken off). Across three experiments using a yes/no recognition paradigm, the presence of a mask was orthogonally manipulated at the study and the test phases. All data showed no evidence of matching effects. In Experiment 1, the presence of masks either at study or test impaired the correct identification of a target. But in Experiments 2 and 3, in which the presence of masks at study or test was manipulated within participants, only masks presented at test-only impaired face identification. In these conditions, test expectations led participants to use similar encoding strategies to process masked and unmasked faces. Across all studies, participants were more liberal (i.e., used a more lenient criterion) when identifying masked faces presented at the test. We discuss these results and propose that to better understand how people may identify a face wearing a mask, researchers should take into account that memory is an active process of discrimination, in which expectations regarding test conditions may induce an encoding strategy that enables overcoming perceptual deficits.

## Introduction

One of the public health guidelines established as a response to the current coronavirus outbreak was the use of masks that cover the bottom half of an individual’s face. Here, we draw on the memory processes involved in facial recognition to examine how the occlusion of the bottom half of a face impacts facial recognition and target identification.

Before the COVID-19 pandemic, researchers had already addressed how facial occlusions—the use of sunglasses, hats/caps, scarfs, beards, medical masks, and religious veils—impact targets’ recognition/identification (e.g., Davies & Flin, 1984; Hockley et al., 1999; Mansour et al., 2017; Nguyen & Pezdek, 2017; Righi et al., 2012; Terry, 1994). All these forms of facial occlusion appeared to deteriorate the recognition of individuals’ identities (e.g., Davies et al., 1977; McKelvie, 1976; Patterson & Baddeley, 1977).

In line with these studies, Freud et al. (2020) showed evidence indicating that face masks impaired identity recognition, regardless of being present when a face is initially encountered (i.e., at encoding) or during a recognition test. Specifically, in their Experiment 2, Freud et al. (2020) found that the presence of a mask in either the study phase or the test phase equally impaired facial recognition. However, this disturbance did not occur when the faces were inverted, that is when the holistic processing of the faces was already disrupted (e.g., Richler et al., 2011). Thus, masks appeared to impair face recognition by disrupting the holistic processing that sustains our ability to identify or recognize a face.

Yet the effects of occluding a face initially, at the encoding phase, or subsequently, at the test phase, might not be independent. Specifically, the match or mismatch of encoding and test conditions might influence whether occlusions impair recognition. For instance, sunglasses occluding the region of the eyes impair target recognition more when encoding and test conditions mismatch (i.e., when the target is initially seen with glasses and subsequently shown without glasses or vice versa) than when the target is shown in both encoding and test with sunglasses (e.g., Leder et al., 2011; Patterson & Baddeley, 1977; Righi et al., 2012; Terry, 1993, 1994). Also, the presence of occlusions during encoding might be more detrimental than during retrieval, as removing the glasses during testing decreases accurate facial identification more than adding them (Douma et al., 2012). Manley et al. (2019) showed that matching appears to occur for masked faces in a lineup identification paradigm, where they manipulated orthogonally the presence of a facial mask both at encoding (seeing an unfamiliar unmasked face vs. a masked face) and retrieval (identifying an unmasked face vs. a masked face). The identification of a face that had been encoded as a masked faced was higher in a masked-face lineup than in an unmasked-face lineup, showing matching effects.

Only designs that orthogonally manipulate the presence of a mask at encoding and retrieval are able to detect the interplay between encoding and retrieval conditions that is assumed to lead to matching effects. This interplay was first hypothesized within the encoding-specificity framework (e.g., Tulving & Thomson, 1973), assuming that “what is stored is determined by what is perceived and how it is encoded and is stored determines what retrieval cues are effective in providing access to what is stored” (p. 353). Congruent with this hypothesis, research has indicated that memory performance increases when the cognitive processes involved in encoding operations are similar/relevant to those involved in retrieval operations, either because the processing conditions of retrieval and encoding are the same (i.e., transfer appropriate processing; see Franks et al., 2000, for a review) or because the conditions of encoding are relevant to or appropriate for the retrieval strategy (Gardiner et al., 1972; Nairne, 2002). In the recognition of occluded faces, matching effects supposedly occur through the transfer of appropriate processing (see Manley et al., 2019), related to the holistic process imposed by a full face and the featured process imposed by the presence of a mask. The research on transfer of appropriate processing suggests that the impact of a mask on facial recognition also likely depends on the dynamic relationship between encoding and retrieval conditions, so that we should expect to find evidence of matching effects when testing for the impact of masks in face recognition.

The reviewed literature suggests that to better understand the current daily impact of a mask in face recognition, we need more evidence than the one offered by studies focusing on how masks interfere with face perception mechanisms and that did not manipulate the presence of masks in encoding and test phases orthogonally. We need evidence about how masks interfere with memory mechanisms and whether such memory mechanisms may help to overcome deficits promoted by masks interfering with the holistic apprehension of a face. The hypothesis that a matching effect is likely to occur with regard to masked and unmasked faces at encoding and retrieval—faces studied with a mask should be better remembered with a mask than without it—is one example of how a memory mechanism can overcome negative effects of masks on face recognition.

Another example relates to the fact that a “masked-faces” memory context may lead faces to be processed differently. An example is the memory testing effect, showing that the expectations of a memory test can induce the use of different encoding strategies (e.g., Finley & Benjamin, 2012). Knowing in advance that our environment is dynamic and that a specific face can later be met with or without a mask is likely to lead individuals to adapt their encoding strategies. Given that memory is an active process, individuals can exercise strategic control over encoding and recall processes (for a review, see Tullis & Benjamin, 2015,) and adapt their encoding strategies to what they anticipate encountering in the test phase (Benjamin, 2007; Dunlosky and Kane, 2007; Finley et al., 2010; Serra & Metcalfe, 2009). Illustrating this, Finley and Benjamin (2012) induced participants to expect a cued recall test or a free recall test and showed that those who received a test that matched their expectations outperformed those who received a mismatched test. Garcia-Marques et al. (2015) followed up on this hypothesis, showing that experienced retrieval contexts can affect subsequent encoding. That is, the specific requirements of retrieval contexts appear to affect subsequent encoding with consequences for recognition and free recall performance. In their experiments, after learning the structure of the test, participants adopted an encoding strategy that avoided a conceptual-based encoding and instead relied on feature-based encoding, which facilitated performance in the test. Performance at retrieval depends on individuals’ ability to attend, during encoding, to the cues that are relevant to subsequent recognition (e.g., Eysenck, 1979; Geiselman et al., 1986; Jacoby et al., 1979; Nairne, 2002; Roediger & Guynn, 1996). In the case of facial recognition, although holistic processing usually supports it, individuals experience different facial features as varying in their diagnostic value for facial identity apprehension (e.g., Nam et al., 2012; but see Sporer, 1991). Previous research suggests that the region of the eyes has some of the most informative value for facial identification (e.g., Davies et al., 1977; Gosselin & Schyns, 2001; Haig, 1985, 1986; Christie et al., 1981) and that facial identification accuracy is more affected by the omission or alteration of upper facial features (e.g., eyes) than lower facial features (e.g., mouth; see Davies et al., 1977; Sinha et al., 2006). The studies developed by Sadr et al. (2003) clarified that not only the eyes but also the eyebrows are among the most important facial features affecting face recognition, as their participants were better at correctly identifying famous faces lacking eyes than lacking eyebrows. If this is the case, masks do not cover the most diagnostic cues for facial recognition.

Several other studies have shown that, in a mutating memory context, individuals adapt their encoding strategies, impacting their face recognition abilities (e.g., Light et al., 1979; Sporer, 1991; Wells & Hryciw, 1984). For instance, by instructing participants to make attributional judgments to disguised faces, Patterson and Baddeley (1977) obtained an improvement in face recognition when compared with undisguised faces (but see Davies & Flin, 1984, for a null effect). Also, if instructions call attention to the individuality of a face, participants will likely attend to more detailed features of the face (Schwartz & Yovel, 2016; Hugenberg et al., 2010). For example, presenting a person’s name along with a face improves the recognition of that face relative to when no name is presented (Schwartz & Yovel, 2016).

Although test expectations may be provided by task instructions, the most likely is that individuals implicitly appraise the context and create their expectations about the memory environment. Participants in a heterogenous encoding context of masked and unmasked targets are likely to apprehend the mutating features of such environment and encode the targets’ distinctive features more than they would in a homogenous encoding context of solely masked or unmasked targets. Previous research showed that recognition can be modulated by encoding conditions, mostly defined by the composition of study lists—mixed lists (i.e., items with and without a manipulated feature appear intermixed in the same list) are compared with pure lists (i.e., items with and without the manipulated feature appear in separate lists) (Jonker et al., 2014; McDaniel & Bugg, 2008; Mulligan & Peterson, 2015; see McDaniel & Bugg, 2008, for a review). The impact of list composition has been detected in production effects (e.g., MacLeod et al., 2010), generation effects (e.g., Slamecka & Graf, 1978), bizarreness effects (e.g., Einstein & McDaniel, 1987), perceptual interference effects (Nairne, 1988), and picture-complexity-superiority effects (Nguyen & McDaniel, 2015). These effects occur in mixed-list but not in pure-list designs, suggesting that encoding conditions that foster the processing of distinctive features may be able to overcome the negative impact of the disruptions of holistic processing. In line with this, Winograd (1981) showed that when participants focused on the processing of distinctive features between items, their performance in a subsequent facial identification task was better than when participants were not led to focused on those distinctive features.

In sum, although evidence shows that masks interfere with holistic processing, disturbing facial identification/recognition, it is not yet clear the role that encoding and retrieval dynamics might play in this process. To contribute to the clarification of this issue, we tested how features of the memory context intervene in masked faces identification/recognition. We tested for evidence of: (a) matching effects (better performance in matching than mismatching study-test conditions), (b) list composition effects (mixed lists compared to pure lists would make processing of masked and unmasked faces more similar), and (c) effects of previous testing (whether individuals’ prior expectations regarding future test features will guide their encoding).

## Current studies

Our research plan encompasses the development of a set of three independent face recognition experiments, with a design that fully crossed masked versus unmasked conditions, presented at test and study. In Experiment 1, we used pure lists in a between-participants manipulation. Two of these four experimental conditions (the mismatch conditions) are identical to the ones used by Freud et al.’s (2020). By using the full possible combinations of masked vs. unmasked at test or at study, we aim to clarify whether Freud et al.’s (2020) results replicate in a context where participants experience a mutating environment, and whether the effect is sustained by two simple main effects or instead emerges from an interaction between study and test conditions. We expected to find that masks generally impair target identification/recognition, given the known interference with holistic perceptual processing, However, memory processes can also interfere with performance in a different way. Thus, we expected that masks promoted less impairment if they were present both at study and test, suggesting that when individuals study a face with a mask, they will subsequently better recognize the upper facial region (i.e., a matching effect).

Experiments 2 and 3 tested whether the same interplay between study and test conditions occurred when masked faces were mixed with unmasked faces, that is in a within-participants design with mixed lists. We assumed that in this memory context participants would better attend to distinctive features of the upper half of the face, independently of the face wearing a mask or not. If that is the case, the presence of a mask at encoding should not deteriorate participants’ memory performance (measured by memory sensitivity). This memory performance should become more dependent on the features of the stimuli that better represent a “distinctive” cue (i.e., a cue that is uniquely associated with the to-be-remembered item; see Roediger & Guynn, 1996), namely the region of the eyes.

In Experiment 3, we directly tested the relevance of test expectations offered by instructions. To do so, we compared conditions where the instructions directed individuals’ attention to features that are likely to change between the study and the test phases (i.e., in the test phase the same face could appear with or without a mask) with conditions where instructions did not direct attention to any particular features.

We tested our hypotheses by using signal detection theory (SDT) and thus assessing the sensitivity (*d’*) and bias or decision criterion (*c*) indexes. According to SDT (e.g., Kadlec, 1999; Kellen et al., 2021; Lockhart & Murdock, 1970; Van der Kellen et al., 2008), better accuracy is defined as a higher sensitivity (*d*’) of target identity, and response caution is defined by using a more conservative criterion in making a positive identification.

For the between participants design, we expected differences in *d’* both in the study and the test conditions, in such that unmasked faces would always be better identified than masked faces, regardless of being presented at study or test. The matching hypothesis would be translated into a specific interaction between the study and test masked versus unmasked conditions, suggesting that performance would be higher for matching conditions than for mismatching conditions. The same would be less likely to occur with a mixed list design in Experiments 2 and 3. If, as expected participants use the same diagnostic features to similarly encode masked and unmasked faces, no main effect of study conditions should emerge, and matching effects should be less likely to occur. In Experiment 3, we expect to clarify the role that the instructions that explicitly direct attention to the mutating features of the environment play in preventing the impact of a mask at encoding.

Differences between the decision criterion (*c*) in each experimental condition can occur either because the presence of a mask reduces or increases the likelihood of participants saying they recognize a face (positive responses). In the first case, responses should show that participants were more cautious (higher *c*) in providing an identification when the face is wearing a mask or more lenient (lower *c*) in providing an identification when the face is not wearing a mask. Thus, as an exploratory hypothesis, we also analyzed whether mixed lists induced more lenience, as a result of participants having followed an encoding strategy more independent of the presence of a mask.

## Experiment 1

### Participants

A sample of 169 Portuguese students (82% women) with a mean age of 21 years old (SD = 4.89; range 18–27) participated in this study in exchange for credits in an introductory psychology course. For power analysis, we relied on Shapiro and Penrod’s (1986) meta-analysis, which reported an effect size for correct identifications (hits) of disguised faces of *d* = 0.71 and considered an effect size of *f* = 0.25. Power analysis using G*Power (Faul et al., 2007) suggested the need for at least 128 participants to detect an effect size *f* = 0.25, 80% power and α = 0.05, regarding the detection of the two main effects and the interactions associated with the planned between-participant design (study list: unmasked face vs. masked face x test list: unmasked face vs. masked face).

### Design

This experiment has a between participants 2 × 2 factorial design, having as independent variables face type at study (masked vs. unmasked) and face type at test (masked vs. unmasked).

### Materials

**Fig. 1:**
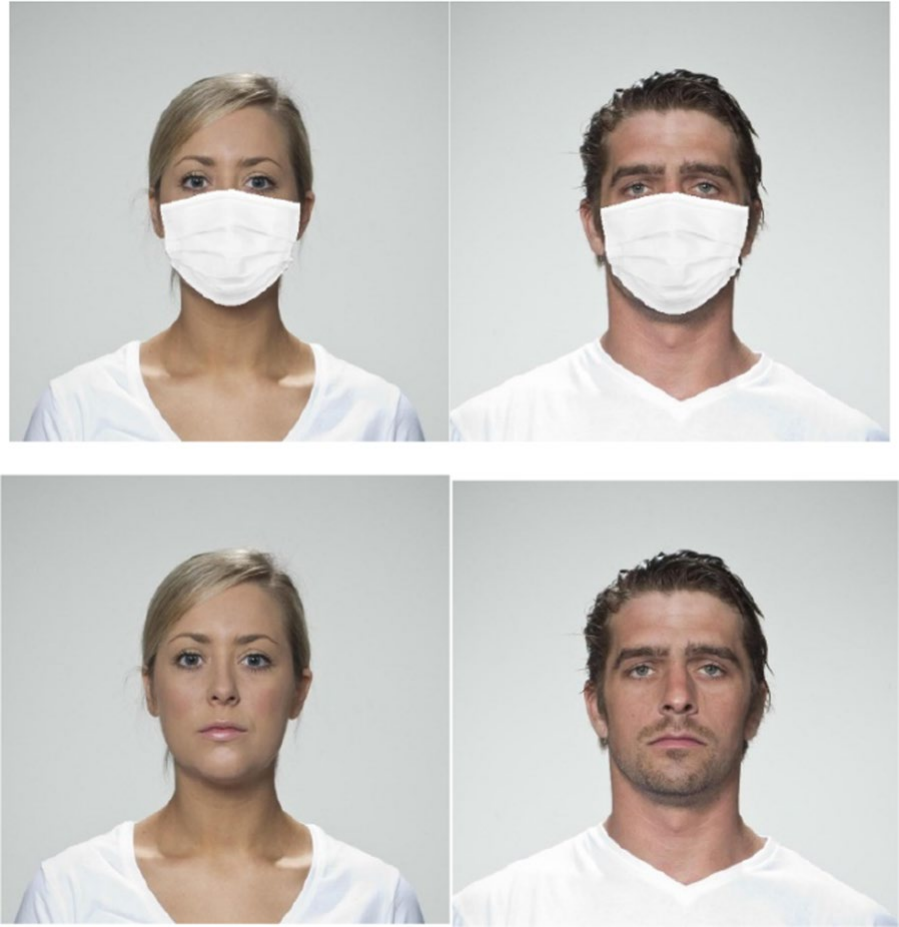
Examples of the Materials used in all Experiments. *Note.* Full face stimuli were extracted from the Face Research Lab London Set (DeBruine & Jones, 2017) Face masking was implemented using the OpenCV v3.4.2 and dlib v19.19 modules within a Python 3.7 environment

The full-face stimuli were extracted from Face Research Lab London Set (DeBruine & Jones, 2017). Thirty-two colored photographs of faces (16 men, 16 women) portraying neutral expressions were selected from the central gaze directions set. The faces selected had similar ages and similar hairstyles (see Fig. 1).

Face masking was implemented using the OpenCV v3.4.2 and dlib v19.19 modules within a Python 3.7 environment. A masked face version of each face image was created by overlaying an edited image of a medical-looking face mask (retrieved from Google Images) onto each face image. Because faces differed in size and structure, we developed a program that dynamically resized and fit the mask image to a fixed configuration of facial landmarks defining the facial region typically covered by a face mask. To address variations in facial shape, each face’s landmarks was dynamically determined by a histogram of oriented gradients (HOG)-based face detector (Dalal & Triggs, 2005) pretrained on a large set of faces under highly variable conditions of expression and environmental factors (300-W database; Sagonas et al., 2016).

At study, all the 32 faces were presented either with a mask (pure masked list) or without a mask (pure unmasked list). For the test phase, half of the studied faces were presented (i.e., old items). For counterbalancing these materials, each gender set of 16 faces was randomly divided into two subsets of eight faces. The test lists either presented one or the other subset. The faces used in the test phase were the exact same faces that had been studied but they could be presented as they had been studied (i.e., masked or unmasked) or with masks added or removed, relative to the study phase. A total of 20 additional faces taken from the same database (10 men, 10 women) were chosen to be randomly presented in the test phase as “new” faces.

### Procedure

Participants were invited to take part in a face memory study, and after informed consent was obtained, they accessed a link to a *Qualtrics survey* that supported the experimental procedure and guaranteed the equal distribution of participants to the four experimental conditions, defined by the type of face studied (unmasked face vs. masked face) and used at test (unmasked face vs. masked face). The instructions stressed that in the memory test, participants should recognize not the photograph but the person in it: *“Try to remember the person behind the mask* *to be able to correctly recognize her or him*.” In the study phase, each participant attended to 32 faces randomly presented at the center of the screen. For half of the participants, those faces belonged to a pure list of unmasked faces, and for the other half the faces belonged to a pure list of masked faces. Each face was shown for 10 s. Then, participants performed a filler perceptual task for 15 min (estimating the width or the length of an image) in order to displace the content from working memory. In the test phase, participants saw 16 studied and 20 new faces, presented randomly at the center of the screen. These faces either matched or mismatched the study mask condition. On each screen, below each face, there were presented two affirmations: “I recognize the person” and “This is a new person.” Participants selected the one that better represented their answer. At the end, participants provided their demographic data (age and gender) and were thanked for their participation.

### Dependent measures

The proportions of correct face recognition (hits) and incorrect positive responses (false alarms; FAs) were calculated for each participant. These were used to calculate *d*′ as an index of sensitivity and *c* as an index of general response tendencies (see, for example, Kadlec, 1999).

## Results

We used a 2 × 2 between-participants ANOVA to analyze the proportion of hits, sensitivity (*d*′), and bias (*c*). Post hoc analysis supporting the interpretation of the interactions relied on Tukey statistics.

### Proportion of correct identifications (hits)

Although faces studied without a mask were better identified than faces studied with a mask (*M* = 0.61, *SE* = 0.02 and *M* = 0.57, *SE* = 0.02, respectively), this difference did not reach conventional levels of significance, *F*(l, 164) = 3.26, *MSE* = 0.09, *p* = 0.068, *η*_p_^2^ = 0.02. Neither a main effect of test condition, *F*(l, 164) = 2.44, *MSE* = 0.06, *p* = 0.129, nor the interaction, *F*(l, 164) = 0.01*, n.s*.^1^ were significant.

### Sensitivity (d′)

**Fig. 2:**
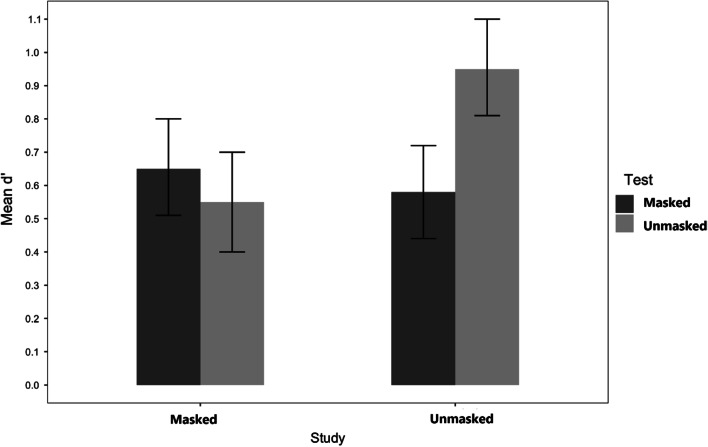
Experiment 1: Sensitivity d’. *Note*. Mean response sensitivity for faces studied with and without a mask and subsequently tested with or without a mask, in Experiment 1

In this analysis, only the study conditions showed a reliable main effect, *F*(l, 164) = 4.97, *MSE* = 1.15, *p* = 0.027, *η*_p_^2^ = 0.029, occurring because faces studied with a mask were less discriminable than faces studied without a mask (*M* = 0.60, *SE* = 0.05 and M = 0.77, *SE* = 0.05, respectively). In the test phase, although results suggested that masked faces were less accurately identified than unmasked faces (M = 0.62, *SE* = 0.05 and *M* = 0.75, *SE* = 0.05, respectively), this difference did not reach significance, *F*(l, 164) = 3.28, *MSE* = 0.75, *p* = 0.072, *η*_p_^2^ = 0.02. However, test and study conditions interacted, *F*(l, 164) = 10.39, *MSE* = 2.40, *p* = 0.002, *η*_p_^2^ = 0.06,. The pattern of the study by test interaction suggests that a matching effect was at work (see Fig. 2). Faces studied without a mask were better recognized without it (*M* = 0.95) than with it (*M* = 0.58), *t*(165) = 3.60, *p* = 0.002, *d* = 0.77. Faces studied with a mask were better recognized with a mask (*M* = 0.65, *SE* = 0.08) than without it (*M* = 0.54, *SE* = 0.07), although this comparison was not significant, *t(*164) = 0.99*, n.s.*

### Criterion (c)

Results indicated that the study conditions did not directly impact individual response tendencies. *F*(l, 164) = 0.60, *n.s*. However different response tendencies were developed in the test phase, *F*(l, 164) = 8.05, *MSE* = 1.24, *p* = 0.005, *η*_p_^2^ = 0.05, showing that participants were more lenient (lower *c*) in their identifications of faces tested with a mask than without a mask (*M* = 0.01, *SE* = 0.04 and *M* = 0.18, *SE* = 0.04, respectively).

**Fig. 3:**
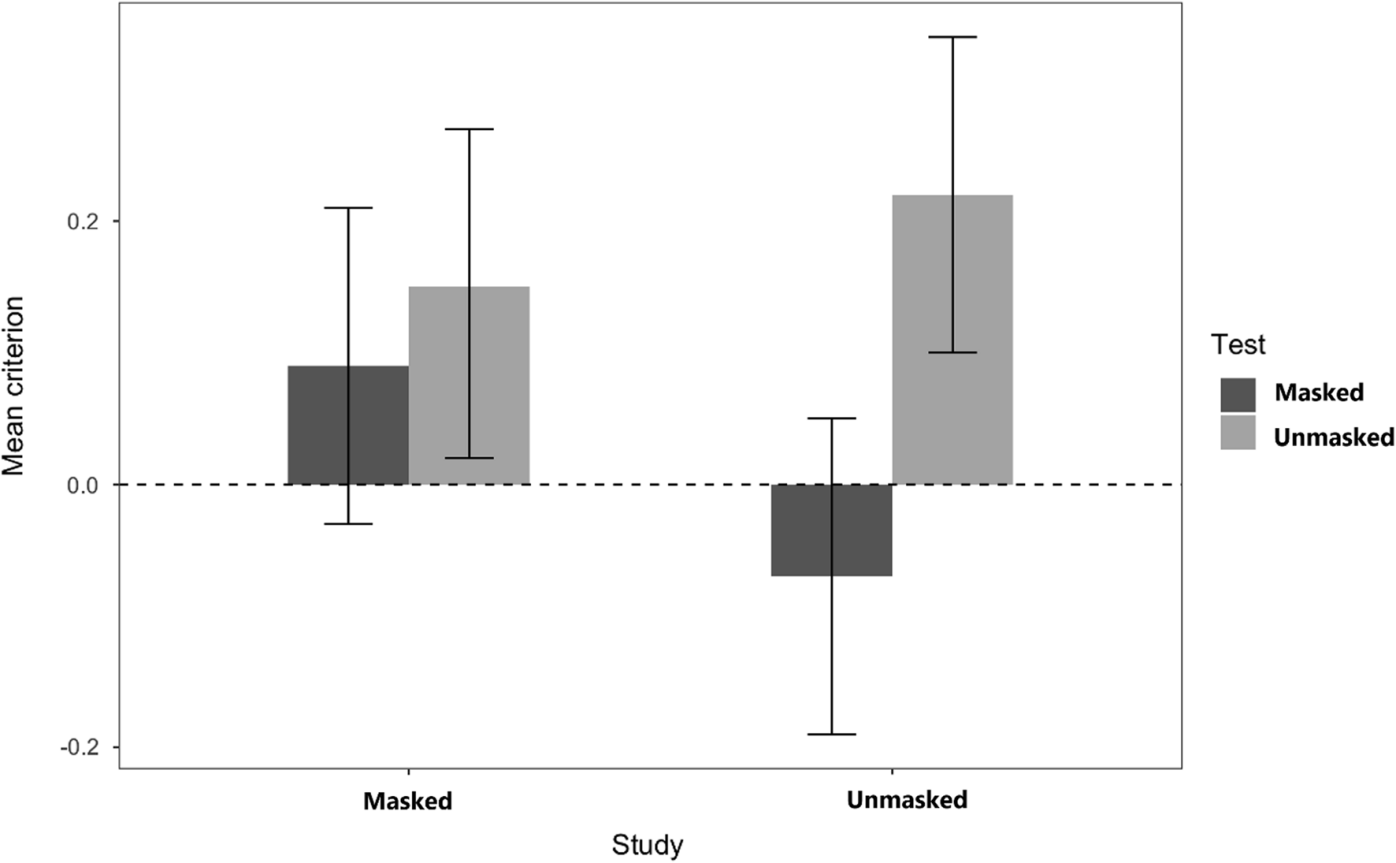
Experiment 1: Criterion c. *Note.* Mean response criterion used for all participants regarding faces studied with and without a mask and subsequently tested either with or without a mask (Experiment 1)

The main effect of test condition was qualified by the study condition (see Fig. 3), and this significant interaction, *F*(l, 164) = 3.90, *MSE* = 0.60, *p* = 0.049, *η*_p_^2^ = 0.02, occurred because the effect of the test condition was clear for faces studied without a mask [*t*(165) = 3.44, *p* = 0.004, *d* = 0.74] but not significant for faces studied with a mask [*t*(164) = 0.06, *n.s*.].

## Discussion Experiment 1

The results of Experiment 1 indicate that, as expected, the presence of a mask at study interferes with subsequent levels of recognition/identification of a face. This effect is better detected with *d’* than with a simple count of *hits* (correct identifications) because *d’* considers parameters that allow for an estimation of the ability to discriminate between signal and noise during the test phase. Data also suggest that a mask at test appears to interfere with levels of recognition, although only by interacting with study conditions, because there was no clear main effect of test condition. These results are partially consistent with Freud et al.’s (2020) conclusions about masks interfering with encoding and retrieval processes. In fact, sensitivity data replicated the results they had obtained in their second experiment because when comparing only conditions with a mismatch between study and test conditions (masked-unmasked versus unmasked-masked), they obtained no differences between conditions. However, the results obtained by Freud et al. (2020) might be explained by their approach of isolating the two cells of a full design where the study and test conditions interact. Our data suggest that such an approach might not be optimal to fully understand the impact of masks in face recognition.,

Regarding the *d’* index, and contrary to what was expected, the interaction between study and test conditions did not fully cross. As such, rather than documenting better performance in matching study-test conditions, the interaction may have emerged only because it was easier for those in unmasked-unmasked conditions to identify a target, than in all the other 3 conditions.

The *c* index of the SDT approach also clarifies that participants are more lenient in offering positive identifications of a masked face than an unmasked face. However, the effect occurs more clearly if at study the faces were seen without a mask. One reason for this to occur is that by continuing to rely on the holistic processing that supported encoding, participants projected their memories over all the masked faces they saw at test. If that is the case, the same results should not be expected in the within-participants design of our next studies because the use of such a strategy is less likely to occur in a context where encoding strategies are not homogeneous, and a single strategy does not serve well all the stimuli encountered. The strategies that are adapted to each type of stimuli tend to work better when isolated in between-participants designs (see Forrin et al., 2016).

## Experiment 2

### Participants

A priori power simulations were conducted using G*Power for analysis the design with a repeated-measures ANOVA. The results suggested that at least 23 participants were necessary to detect with 90% power both main effects and an interaction in a within-participants design, assuming α = 0.05 and the correlation among repeated measures is 0.50. A sample of 34 Portuguese students (83% women) with a mean age of 23 years old (SD = 7.50; range 17–35) participated in this study in exchange for credits in an introductory psychology course.

### Design

This experiment has a within participants 2 × 2 factorial design, having as independent variables face type at study (masked vs. unmasked) and face type at test (masked vs. unmasked).

### Materials

We used the same materials as in Experiment 1. Study lists contained 32 faces, 16 of which masked and the other 16 unmasked. For counterbalancing the faces’ presentation, two sublists were created, with 16 faces of each gender each. The faces in each sublist were randomly divided into two subsets of eight faces each, and the subsets presented with a mask in one list were presented without a mask in the other list. Four test lists were created having each subset divided in two halves, one presented with a mask and the other without a mask. Thus, targets in the test phase were the exact same faces that had been studied but they either appeared as they had appeared during study (masked or unmasked) or now the mask was removed or added. A total of 20 additional “new” faces were added to the test list.

### Procedure

Participants were invited to take part in a memory study via a Qualtrics survey. After informed consent was obtained, participants started the task. This task was similar for participants and defined the four conditions of the within-participants design; 2 (Study: unmasked face vs. masked face) × 2 (Test: unmasked face vs. masked face) conditions.

In the study phase, participants saw a mixed list of masked and unmasked faces, presented randomly at the center of the screen. The instructions stressed (a) the heterogeneous features of the list (the presence of male and female and faces with and without a mask) and (b) that in a future memory task, participants would have to recognize not the photograph but the person in it.

Study procedure was similar to the procedure used in Experiment 1. In the test phase, participants saw the studied items randomly mixed with the set of “new” items. The studied items could be the exact same photographs (i.e., either wearing a mask or not, as in the study phase) or a photograph of the same person they had seen during study but now seen with a mask added or removed. Half of the new faces were unmasked and the other half were masked. The different sets of materials were counterbalanced across the participants. The instructions stressed the need to say “yes” if they “*saw the person on the list of people previously presented”* independently of having or not having a mask now or before.

Finally, we collected information on the age and gender of the participants and thanked them for their participation.

### Dependent measures

**Fig. 4:**
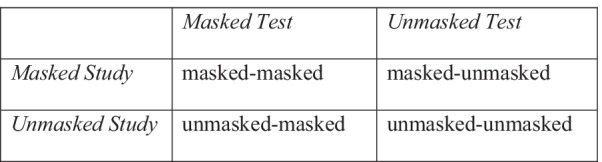
A depiction of the within-participants conditions used in Experiment 2. *Note*. For each of these conditions, hits and false alarms (FAs) were independently calculated for each participant, to support the calculation of the *d’* and *c* indexes

For each participant, the proportions of correct identifications (hits) and incorrect positive identifications (FAs) were calculated for each of the four cells of the within-participant design (see Fig. 4).

The within nature of the design imposes special conditions for calculating SDT indexes with independent values.^2^ For that reason, for each participant, the set of new unmasked faces and masked faces were randomly separated into two halves (the randomization process guaranteed that each half had a different set of faces). The randomization was implemented using R v4.0.2 (R Core Team, 2020). The four resulting sets were associated with one of the cells of the design, in such that incorrect positive responses (FAs) for each of those represented the false alarm values of each cell: masked-masked FA, unmasked-masked FA, masked-unmask FA, unmasked-unmasked FA. These were used to calculate *d*′ as an index of sensitivity and *c* as an index of general response tendencies for faces tested with and without a mask.

## Results

To test our hypotheses, we used repeated-measures ANOVAs with encoding (Study) and retrieval (Test) conditions defined as within-subject factors. For post hoc analysis of the interactions, we used Tukey statistics.

### Proportion of correct identifications (hits)

The analysis of the number of correct identifications showed only a reliable main effect of the test condition, *F*(l,33) = 8.28, *MSE* = 0.04, *p* = 0.007, *η*_p_^2^_=_ 0.20. The effect suggested that at test the participants identified more masked faces (M = 0.68, *SE* = 0.03) than unmasked faces (*M* = 0.57, *SE* = 0.03). Neither a main effect of study condition, F(l,33) = 1.07, *MSE* = 0.35, *p* = 0.309, *η*_p_^2^_=_ 0.031, nor an interaction between study and test conditions were significant, *F*(l,33) = 1.63, *MSE* = *0.04*, *p* = 0.211, *η*_p_^2^ = 0.05.

### Sensitivity (d′)

**Fig. 5:**
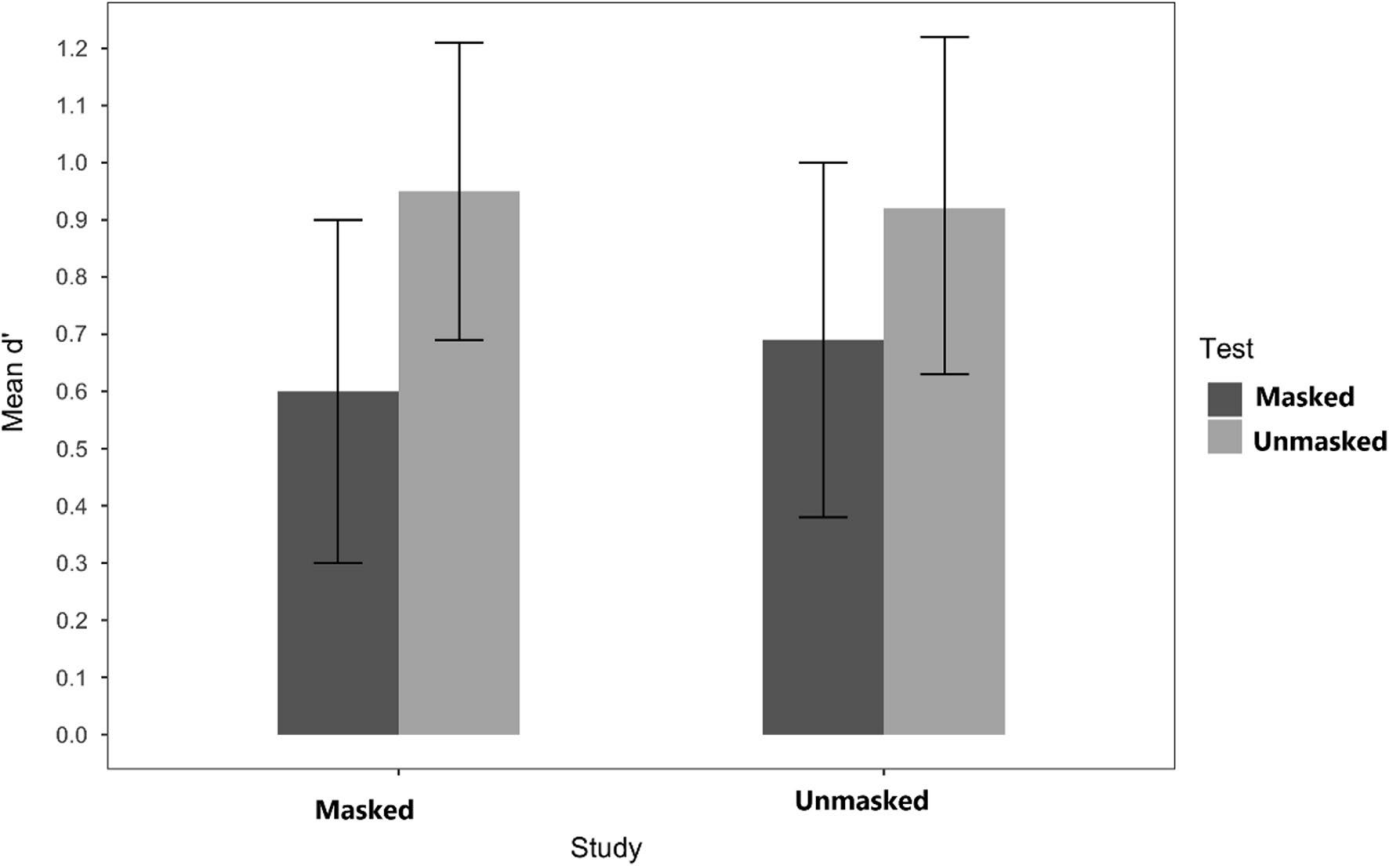
Experiment 2: Sensitivity d’. *Note.* Mean response sensitivity for faces studied with and without a mask and subsequently tested either with or without a mask (Experiment 2)

The scores of how well individuals could discriminate a studied face presented with or without a mask showed no significant effects. The difference suggesting that, at test, participants discriminate better between studied and unstudied faces when they are unmasked at test (*M* = 0.94, *SE* = 0.10) than when they are masked (*M* = 0.65, *SE* = 0.11) did not achieve conventional levels of significance, *F*(l, 33) = 3.67, *MSE* = 0.77, *p* = 0.064, *η*_p_^2^ = 0.10. There was also a null effect of study conditions, *F*(l, 33) = 0.05, *n.s.,* suggesting that participants discriminate previously presented faces equally well when they had appeared masked during study (*M* = 0.78, *SE* = 0.10) and when they had appeared unmasked during study (*M* = 0.81, *SE* = 0.10). The study by test interaction was not significant, *F*(l, 33) = 0.16, *n.s.* (see Fig. 5). The difference between the two mismatch conditions was also not reliable, (*M*_*Unmasked-Masked*_ = 0.69, *SE* = 0.15 and *M*_*Masked-Unmasked*_ = 0.95, *SE* = 0.13); *t*(33) = 1.32, *p* = 0.554.

### Criterion (c)

**Fig. 6:**
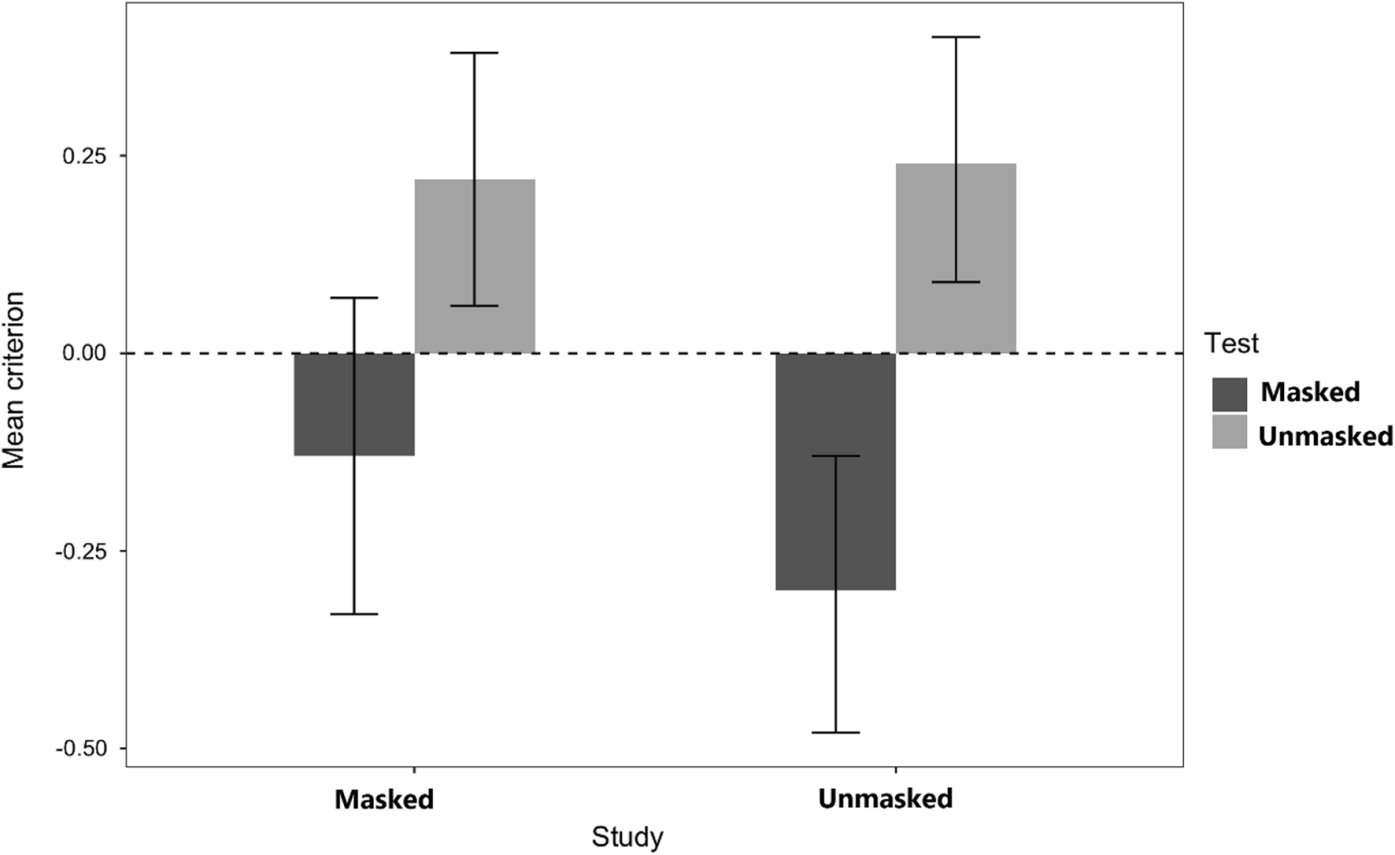
Experiment 2: Criterion c. *Note*. Mean response criterion used by all participants regarding faces studied with and without a mask and subsequently tested with or without a mask (Experiment 2)

The analysis of the criterion scores indicated that in the test phase, participants were less cautious in offering a positive identification of a masked face (*M* = -0.23, *SE* = 0.08) than of an unmasked face (*M* = 0.23, *SE* = 0.08), leading to a main effect of test, *F*(l,33) = 32.84, *MSE* = 0.21, *p* = 0.001, *η*_p_^2^ = 0.50. The main effect of study condition was not significant, *F*(l, 33) = 1.36, *MSE* = 0.15, *p* = 0.252 *η*_p_^2^ = 0.04, as study condition did not influence to individuals’ response tendencies (*M*_Masked_ = 0.05, *SE* = 0.08 vs. M_Unmasked_: = − 0.03, *SE* = 0.07). The interaction between study and test (see Fig. 6) was also not reliable, *F*(l, 33) = 2.92, *MSE* = 0.12, *p* = 0.097, *η*_p_^2^ = 0.08.

## Discussion Experiment 2

The results diverge from those obtained in Experiment 1. Here, study conditions (masked or unmasked) were not relevant for subsequent face recognition/identification. But participants’ number of correct responses was impaired when at test the target face was presented with a mask.

The results also diverge from those obtained in Experiment 1 in an unexpected way. The interaction between study and test was not replicated. Here we would have expected to find a clear matching effect because matching advantages are most reliably obtained in within-participants designs (see, for instance, Dewhurst & Brandt, 2007; Dewhurst & Knott, 2010; Mulligan & Lozito, 2006). The fact that we did not find such evidence may have been because in this specific heterogenous setting, participants’ retrieval might not have relied on the same processes that supported their encoding. A possible cause for this is that the presence of unmasked faces in the list of masked faces could have imposed holistic processing of all the faces in the list.

Importantly, when comparing the two mismatching cells of the design (masked-unmasked; unmasked-masked), the results converge with those obtained by FreudFreud et al.’s (2020). Like the authors suggested, these results might have occurred because masks interfered with the holistic processing. However, Experiment 2 clarifies that by looking at the complete design we can infer that the presence of unmasked faces in the mixed lists also interfered with how masked faces were processed.

In Experiment 1, we offered a likely explanation for the increase in response lenience of participants when recognizing a masked face. Congruently, we expected that the qualification of a test main effect by study conditions would not occur in a within-participants experiment. Results supported those expectations.

In Experiment 3, we tested the reliability of these results and accessed their dependence on a priori knowledge about the test format. Thus, Experiment 3 replicates Experiment 2 and adds a condition in which no expectations were created relative to the mutability of the environment (i.e., the same face could be presented with or without a mask at test).

### .

## Experiment 3

### Participants

Participants were recruited online through the Prolific platform. A total of 70 participants, with a mean age of 26 (S*D* = 5.70; range 18–34), 58% were female, were distributed by two experimental conditions, defined by the instructions provided. We calculated post hoc power that could be associated with the possibility that instructions qualify the main effects and interaction between study and test within experimental conditions. The results suggested that the power associated with this moderation would be higher than 0.90.

### Design

This experiment has within-participants manipulations of 2 (Study: unmasked face vs. masked face) × 2 (Test: unmasked face vs. masked face) to which a between participant independent variable was added: type of instructions (stressing or not the mutability of the environment).

### Procedure

Participants were randomly assigned to one of the two instructions groups. Within those groups, all participants performed the same task, which defined the four conditions of the within-participants design.

One group of participants received instructions stressing that a face studied with a mask could be subsequently presented either with or without a mask, and a face studied without a mask could subsequently be presented with or without a mask. As such, they should *try to remember the person behind the mask to be able to correctly recognize the person in the memory task.* The other group of participants received instructions that just stated the need to study the faces to subsequently perform a memory test.

### Dependent measures

We used the same measures used in Experiments 1 and 2. FAs for each cell of the design were created by the same random distribution to different sets of new faces that was used in Experiment 2.

## Results

To test our hypotheses, we used mixed ANOVAs with encoding (Study) and retrieval (Test) conditions defined as within-subject factors and instructions as a between factor. For post hoc analysis of the interactions, we used Tukey statistics.

### Proportion of correct identification (hits)

The analysis of the proportion of correct identifications indicated that the two main effects were significant. The main effect of study, *F*(l, 67) = 4.71, *MSE* = 0.13, *p* = 0.033, *η*_p_^2^ = 0.07, occurred because unmasked studied faces were more positively identified (*M* = 0.65) than masked studied faces (*M* = 0.60). The main effect of test, *F*(l, 67) = 13.36, *MSE* = *0*.41*, p* < 0.001 *η*_p_^2^ = 0.17, occurred because masked tested faces were more positively identified (*M* = 0.68. *SE* = 0.02) than unmasked tested faces (*M* = 0.59, *SE* = 0.02). Additionally, the interaction between test and study was significant, *F*(l, 67) = 5.71, *MSE* = 0.16, *p* = 0.020, *η*_p_^2^ = 0.08 This interaction clarified that better identification of a target using a mask at test occurred for faces that were previously studied without a mask (*M*_*Masked*_ = 0.70: *SE* = 0.02 vs *M*_*Unmasked*_ = 0.58, *SE* = 0.02; *t*(*67*) = 4.36, *p* < 0.001, *d* = 0.78, but not for faces studied with a mask (*M*_*Masked*_ = 0.61, *SE* = 0.03 vs M_*Unmasked*_ = 0.58, *SE* = 0.03), *t*(67) = 0.13, *n.s*. Instructions did not qualify any of the effects described above, all *Fs* < 1, [Study x Instructions: *F*(l, 67) = 0.44; Test x Instructions, *F*(l, 67) = 0.59; 3-way, *F*(l, 67) = 0.22, all *n.s*.)].

### Sensitivity (d′)

**Fig. 7:**
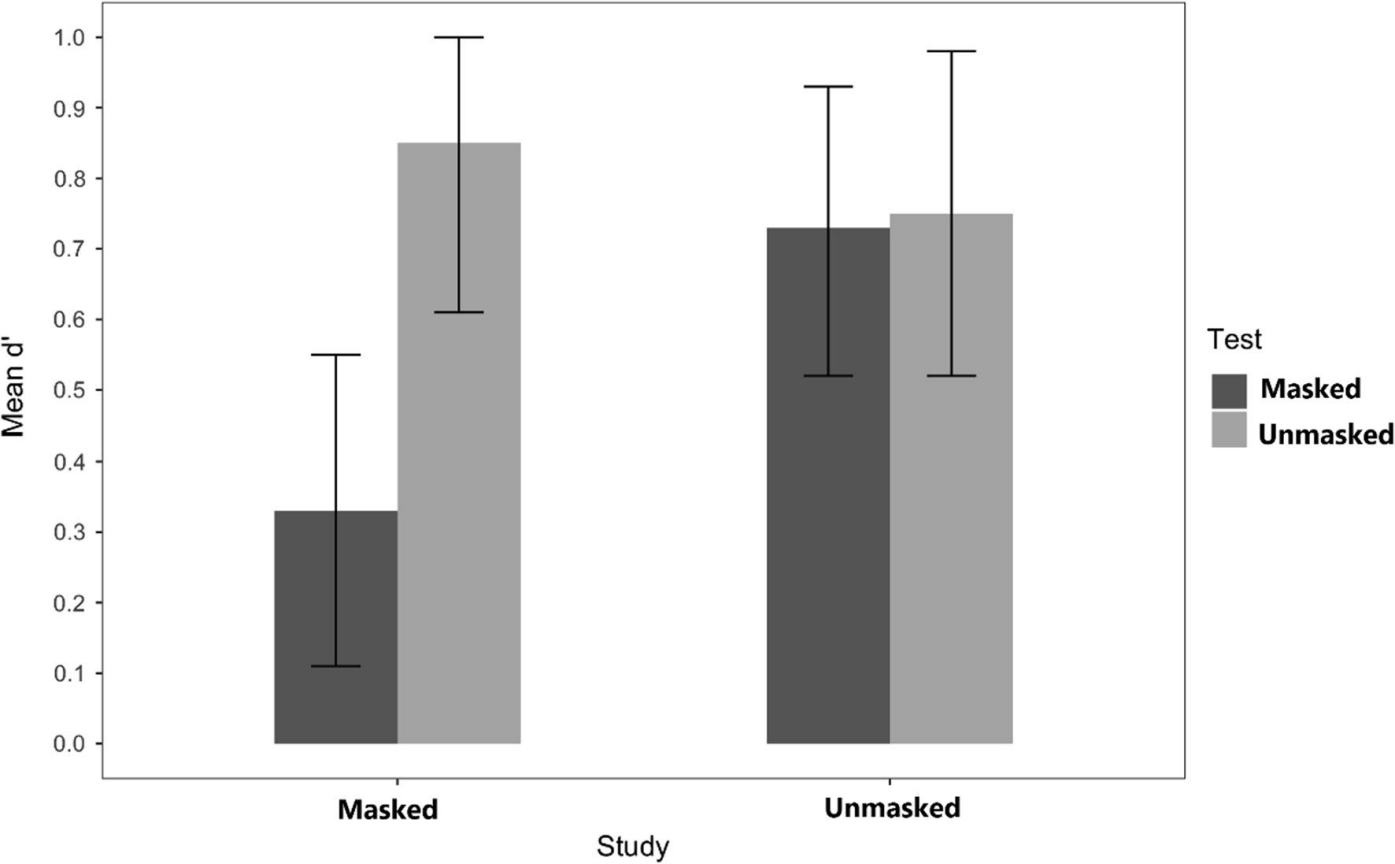
Experiment 3: Sensitivity d’. *Note.* Mean response sensitivity for faces studied with and without a mask and subsequently tested either with or without a mask (Experiment 3)

Replicating Experiment 2, we found no significant main effect of study *F*(l, 67) = 2.26, *MSE* = 1.45, *p* = 0.137, *η*_p_^2^_=_0.03, (*M*_*Masked*_ = 0.59, *SE* = 0.10, *M*_*Unmasked*_ = 0.74, *SE* = 0.09) but a main effect of test emerged, *F*(l, 67) = 10.0.42, *MSE* = 4.95, *p* = 0.002, *η*_p_^2^_=_0.13, with performance being better for unmasked faces (*M* = 0.80, *SE* = 0.09) than for masked faces (*M* = 0.53, *SE* = 0.09). Additionally, the interaction between test and study was also significant, *F*(l, 67) = 8.41, *MSE* = 4.14, *p* = 0.005, *η*_p_^2^ = 0.11. This interaction (see Fig. 7) appeared to occur because the better identification at test of an unmasked face over a masked face was not obtained for unmasked studied faces, *t*(67) = 0.02, *n.s*, but only for masked studied faces, *t*(67) = 4.71, *p* < 0.001, *d* = 1.00. Also the two cells of the mismatched conditions (*M*_*Masked-Unmasked*_ = 0.85, *SE* = 0.12 and *M*_*Unmasked-Masked*_ = 0.75, *SE* = 0.11) did not differ significantly, *t*(67) = 0.97, *n.s*.

None of the instruction effects were significant. The study by instructions interaction, was not significant, *F*(l, 67) = 1.02, *MSE* = 0.66 *p* = 0.315 *η*_p_^2^ = 0.01, and all other effects had *Fs* < 1 [Main effect *F*(l, 67) = 0.01; Test x Instructions, *F*(l, 67) = 0.08; 3-way, *F*(l, 67) = 0.46]. Nevertheless, because the pattern of results did not fully replicate the results of Experiment 2, we performed a simple analysis to test for the replication.

For the group that received instructions stressing the mutating environment (as in Experiment 2), results replicated more closely the results of Experiment 2—no reliable main effect of study, *F*(l, 39) = 3.09, *MSE* = 0.67, *p* = 0.070, *η*_p_^2^ = 0.08, and no significant interaction, *F*(l, 39) = 2.97, *MSE* = 0.486, *p* = 0.090, *η*_p_^2^ = 0.070. Contrary to what occurred in Experiment 2, the main effect of test here achieved significance, *F*(l, 37) = 9.29, *MSE* = 3.75, *p* = 0.004 *η*_p_^2^ = 0.19.

For the group that received instructions that did not direct attention to the mutating environment, results were slightly different. Both the main effects of study, *F*(l, 28) = 0.11, *n.s.*, and test, *F*(l, 28) = 2.98, *MSE* = 1.77, *p* = 0.100 *η*_p_^2^ = 0.09, failed to reach significance. Only a reliable interaction between study and test emerged, *F*(l, 28) = 5.44, *MSE* = 2.72, *p* = 0.027 *η*_p_^2^ = 0.16. This interaction occurred because masks were more detrimental to recognition when presented both at study and at test (*M*_*Masked-Masked*_ = 0.36, *SE* = 0.13) than in the other three conditions, all *p* < 0.001. The three other conditions (*M*_*Masked-Unmasked*_ = 0.92, *SE* = 0.17; *M*_*Unmasked-Masked*_ = 0.72, *SE* = 0.16; *M*_*Unmaksed-Unmasked*_ = 0.66, *SE* = 0.16) did not differ significantly, all *p* > 0.30.

### Criterion (c)

**Fig. 8:**
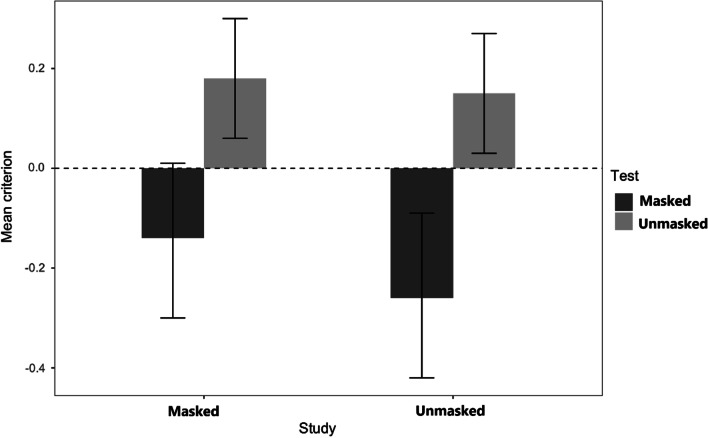
Experiment 3: Criterion c. *Note*. Mean response criterion used for all participants regarding faces studied with and without a mask and subsequently tested either with or without a mask (Experiment 3)

For criterion scores, only the test main effect was significant. The main effect of study*, F*(l, 67) = 2.24, *MSE* = 0.37 *p* = 0.139, *η*_p_^2^ = 0.03, shows that participants were equally liberal in providing positive identifications of masked studied faces (*M* = -0.05, *SE* = 0.06) than unmasked studied faces (*M* = 0.02, *SE* = 0.06). The main effect of test, *F*(l, 67) = 36.29, *MSE* = *38*.06, *p* < 0.001, *η*_p_^2^_=_ 0.36, occurred because participants were more liberal in providing positive identifications of faces masked at test (*M* = -0.20, *SE* = 0.07) than faces unmasked at test (*M* = 0.17, *SE* = 0.05). The interaction between the two factors (see Fig. 8) was not significant, *F*(l, 67) = 2.75, *MSE* = 0.30, *p* = 0.102, *η*_p_^2^ = 0.04. Once again, the instructions had no effect, the test by instructions interaction was not significant, *F*(l, 67) = 1.38, *MSE* = 0.23 *p* = 0.247 *η*_p_^2^ = 0.02, and all other effects were not significative having an *F* < 1 [Main effect *F*(l, 67) = 0.18; Study x Instructions, *F*(l, 67) = 0.08; 3-way, *F*(l, 67) = 0.03, all *n.s*.].

## Discussion Experiment 3

The results of Experiment 3 partially replicated the results of Experiment 2, showing that in a mixed list context faces that were studied with or without a mask have an equal probability of being subsequently identified. Also, performance was more impaired if, at the test, the target face was presented with a mask than without a mask.

However, the general results diverge from those found in Experiment 2, by adding to them a significant interaction between study and test. Again, the interaction pattern was not one of a matching advantage. Instead, the interaction pattern suggested that faces studied with a mask were the ones that suffered the most from being tested with a mask. An effect that should not have been obtained if transfer of appropriate processing occurred and, therefore, challenges the idea that masked and unmasked faces in this heterogeneous context were encoded similarly.

A simple analysis testing the effect separately in each instruction group allowed us to better understand this interaction. First, because it clarified that when instructions led participants to create expectations regarding test conditions, results fully replicated those of Experiment 2. Second, because it showed that the interaction emerged when no main effects occurred, suggesting that without expectations about test format, a mixed list of masked and unmasked faces did not foster a similar and uniform strategy to encode masked and unmasked faces.

One caveat is that the pattern of the interaction indicates that no encoding strategy of a masked face can facilitate the subsequent identification of the same face when masked at the same level that an encoding strategy of an unmasked face can facilitate the subsequent identification of the same face when masked. Also contrary to what would be expected, faces studied with masks were better identified without masks than with masks. This result is not showing the whole interference effect (Leder & Carbon, 2005): The studied masked faces should suffer the interference of the whole unmasked face presented at test. Performance at test should have been impaired in the masked-unmasked condition as a result of a holistic interference during the test phase.

In general, participants were more lenient in providing a positive identification for faces wearing a mask at test.

## General discussion

In this research, we assessed how memory mechanisms might intervene in the processes by which a face mask can impair face identification/recognition. Although supported by holistic processing, face recognition/identification might also benefit from the transfer of appropriate processing and context features that either decrease or increase the processing of distinctive features. We tested whether these two memory processes might counteract the assumed disruption of holistic processing caused by the addition of a mask to a face.

In three studies, we addressed transfer of appropriate processing translated into matching effects (between study and test) by manipulating orthogonally whether masks were presented at encoding and at retrieval. Based on previous research (see Manley et al., 2019), we expected to obtain recognition/identification matching effects, across the three studies. However, we did not find support for this hypothesis. Although results showed an interplay between study and test conditions, both in pure list conditions (Experiment 1) and in mixed list conditions when instructions did not create expectations about the testing context (Experiment 3), the pattern of this interplay was different from what would be expected by a matching hypothesis. The interaction occurred because masks were more detrimental for recognition when occurring in both study and test conditions.

We also assessed whether expectations about the memory test could induce similar encoding strategies for masked and unmasked faces, expecting that the use of these strategies would be more likely to occur in a mixed list study condition. In accordance with this testing-expectancy hypothesis (e.g., Finley & Benjamin, 2012), we found no impact of study conditions on subsequent performance, in both Experiments 2 and 3. The encoding adaptation to the test occurred for mixed list of materials and not for pure lists of materials. This suggests that the instructions provided were not, by themselves, enough to promote these effects. Maybe experiencing a mixed environment (with masked and unmasked faces) was necessary to direct participants to attend to upper face distinctive features. Although we did not find direct evidence of instruction effects in Experiment 3, a simple analysis suggested that using mixed lists was also insufficient for promoting similar encoding of masked and unmasked faces. To better understand what features of the context more clearly modulate encoding in face study and recognition, future studies should clarify these results.

Across the three experiments, we replicated Freud et al.’s (2020) result showing that a mask equally impaired recognition when it was present only at study or only at test. However, our results clarify that the null difference between these two conditions emerges from different patterns of effects, not allowing the conclusion that masks at study are always detrimental to face identification. Although, as the authors claimed, masks interfered with holistic face processing, impacting face recognition. However, face recognition is also supported by memory processes that can help counteract deficits in holistic processing, as our data suggest.

Face recognition or target identification has been approached in the literature by focusing on face perception mechanisms. However, as face recognition is a memory process it also depends on memory mechanisms, which should be further studied in the face-recognition literature. The studies that already documented that masks interfere with face processing are not informative about how well we will be able to identify a person met with a mask. These studies just tell us that recognition of a face we meet masked will be different from recognition of a face we meet unmasked. For instance, Freud et al. (2020) focused on understanding how a mask disrupts holistic processing, and Dhamecha et al. (2014) and Carragher and Hancock (2020) showed that masks impaired perceptual mechanisms that support the identification of faces in a matching task. However, although performance in a matching task is usually correlated with recognition memory for faces (Burton et al., 2010; Megreya & Burton, 2006), this task minimizes the contribution of memory mechanisms and increases reliance on perceptual mechanisms (Estudillo & Bindemann, 2014; Megreya & Burton, 2008). We believe that to better understand the processes involved in face recognition and identification, we should also attempt to understand how contexts in which a mask usually appears can modulate our memory.

Our current experiments raise two additional topics that also deserve discussion. One is that in our empirical approach we used the exact same photographs of learnt faces at study and at recognition, during the test phase. We believe that this approach was needed because using the same photograph allowed us to better isolate the effects of the presence of masks. However, this approach confounds face/identity recognition with picture recognition. This is a general (and common) limitation in the field of face recognition research because the processes effecting picture recognition and face identity recognition may differ. Nevertheless, in the context of our experiments this may be more problematic because in the matched conditions all the pictorial cues that were evident during study are also available during the test, whereas in the mismatched conditions only some pictorial cues remain (the upper half of the face) while others differ between study and test (the lower half of the face). This can create a confound in the sense that the nature of the task itself might be different for matched and mismatched conditions. Thus, future research would gain by comparing our conditions with condition in which the photographs change from study to test, across all conditions.

The second topic concerns the methodological strategy followed to match a SDT approach to our within-participants design. To properly calculate signal detection indexes, independent FA scores are necessary for each experimental condition. The independence of scores in a typical within-participants design cannot be ensured, which leads some authors to refrain from using SDT in this type of designs (Icht et al., 2020). However, some researchers have argued that some methodological strategies might overcome this problem (e.g., Forrin et al., 2016; Morrell et al., 2002). Those strategies typically imply a planned design using different lists of material (usually different categories, such as animals and objects) to assess independent hits and FA. Our approach in the current studies involved using a different list of stimuli of the same nature (i.e., faces). And because we did not plan this in advance, we strategically created a different list of new unmasked faces and masked faces for each participant by randomly separating the original sets into two halves. Thus, FAs could be reliably associated with a single condition. By doing this at an individual level, we followed a conservative strategy that made those values independent of the specific faces being presented. Future studies should critically assess the merit of the current approach in comparison with other possible strategies.

In sum, in this paper we place the study of masked faces within the theoretical and methodological discussions around the differences typically found between within- and between-participants designs (see Forrin et al., 2016). As in the general literature, our data suggest that the effectiveness of encoding strategies adapted to each type of stimuli are likely better understood in between-participants designs. In those, we can isolate the encoding of each type of stimuli in pure lists. The use of mixed lists is then informative about the interference that different types of materials exert over each other. In a real-life context, this interference is likely to naturally occur, making relevant any attempt to understand how contexts in which a mask usually appears can modulate our memory.

## Data Availability

At request to gmarques@ispa.pt.

## Footnotes

1 *F* values should not be smaller than 1 given that the *F* statistic is a ratio of mean squares. Thus, whenever an *F* value is smaller than 1, it should not be associated with a *p*-value, which theoretically is 1 [see, for instance, Voelkle et al. (2007) for further explanation]. The same is valid for *t* values between -1 and 1.

2 Unlike between-participants designs, a within-participants design tends to rely on a collapsed false alarm rate which creates data interdependence and biases calculations of SDT indexes. Authors who addressed this problem have suggested different methodological strategies to overcome it (e.g., Forrin et al., 2016; Morrell et al., 2002). See our discussion for a more detailed examination of this issue.
